# The Impact of Frailty on Left Ventricle Mass and Geometry in Elderly Patients with Normal Ejection Fraction: A STROBE-Compliant Cross-Sectional Study

**DOI:** 10.3390/jcdd13010050

**Published:** 2026-01-16

**Authors:** Stanisław Wawrzyniak, Ewa Wołoszyn-Horák, Julia Cieśla, Marcin Schulz, Michał Krawiec, Michał Janik, Paweł Wojciechowski, Iga Dajnowska, Dominika Szablewska, Jakub Bartoszek, Joanna Katarzyna Strzelczyk, Michal M. Masternak, Andrzej Tomasik

**Affiliations:** 1 Doctoral School, Medical University of Silesia, Poniatowskiego 15, 40-055 Katowice, Poland; stawaw95@gmail.com (S.W.); julciesla14@outlook.com (J.C.); michal.krawiec@interia.pl (M.K.); d201296@365.sum.edu.pl (M.J.); 2 Department of Cardiology, Specialist Hospital in Zabrze, 41-800 Zabrze, Poland; krystyna.woloszyn@interia.pl; 3 Students’ Scientific Group at the II Department of Cardiology, Faculty of Medical Sciences in Zabrze, Medical University of Silesia, 40-055 Katowice, Poland; mail.schulz.od44@gmail.com (M.S.); s88354@365.sum.edu.pl (P.W.); s88499@365.sum.edu.pl (I.D.); s88468@365.sum.edu.pl (D.S.); s85584@365.sum.edu.pl (J.B.); 4 Department of Medical and Molecular Biology, Faculty of Medical Sciences in Zabrze, Medical University of Silesia, 40-055 Katowice, Poland; jstrzelczyk@sum.edu.pl; 5 Burnett School of Biomedical Sciences, College of Medicine, University of Central Florida, Orlando, FL 32816, USA; michal.masternak@ucf.edu; 6 Department of Head and Neck Surgery, Poznań University of Medical Sciences, 61-701 Poznań, Poland; 7 II Department of Cardiology in Zabrze, Faculty of Medical Sciences in Zabrze, Medical University of Silesia, 40-055 Katowice, Poland

**Keywords:** frailty, frailty phenotype criteria, left ventricular mass, left ventricular geometry, left ventricular remodeling, concentric hypertrophy, concentric remodeling, relative wall thickness, mechanistic

## Abstract

**Background:** There exists some inconsistent evidence on the relationship between altered cardiac morphology, its function, and frailty. Therefore, this study aimed to assess the associations among frailty, lean body mass, central arterial stiffness, and cardiac structure and geometry in older people with a normal ejection fraction. **Methods:** A total of 205 patients >65 years were enrolled into this ancillary analysis of the FRAPICA study and were assessed for frailty with the Fried phenotype scale. Left ventricular dimensions and geometry were assessed with two-dimensional echocardiography. Fat-free mass was measured using three-site skinfold method. Parametric and non-parametric statistics and analysis of covariance were used for statistical calculations. **Results:** Frail patients were older and women comprised the majority of the frail group. Frail men and women had comparable weight, height, fat-free mass, blood pressure, central blood pressure, and carotid–femoral pulse wave velocity to their non-frail counterparts. There was a linear correlation between the sum of frailty criteria and left ventricular end-diastolic diameter (Spearman R = −0.17; *p* < 0.05) and relative wall thickness (Spearman R = 0.23; *p* < 0.05). In the analysis of covariance, frailty and gender were independently associated with left ventricular mass (gender: β of −0.37 and 95% CI of −0.50–−0.24 at *p* < 0.001), the left ventricular mass index (gender: β of −0.23 and 95% CI of −0.37–−0.09 at *p* < 0.001), and relative wall thickness (frailty: β of −0.15 and 95% CI of −0.29–−0.01 at *p* < 0.05; gender: β of 0.23 and 95% CI of 0.09–0.36 at *p* < 0.01). Frailty was associated with a shift in heart remodeling toward concentric remodeling/hypertrophy. **Conclusions:** Frailty is independently associated with thickening of the left ventricular walls and a diminished left ventricular end-diastolic diameter, which are features of concentric remodeling or hypertrophy. This association appears to be more pronounced in women. Such adverse cardiac remodeling may represent another phenotypic feature linked to frailty according to the phenotype frailty criteria.

## 1. Introduction

People worldwide are living longer, and populations are aging. By 2050, the world’s population of people aged 60 years or older will double (2.1 billion). The number of persons aged 80 years or older is expected to triple from 2020 to 2050, reaching 426 million [https://www.who.int/news-room/fact-sheets/detail/ageing-and-health, accessed on 24 June 2023]. Older adults are susceptible to chronic medical conditions, sensory impairments, and geriatric syndromes, very often combined into multimorbidity. This will translate into increased health care burden and increased social, societal, and institutional costs. The better we understand all the pathophysiological complexities of aging, the better we will be able to allocate the resources. It has been previously described that aging and female sex are associated with left ventricular hypertrophy [1,2,3]. Age-related thickening and stiffening of the large arteries, due to collagen and calcium deposition and loss of elastic fibers in the medial layer, are known to induce systolic hypertension [4]. Hypertension, along with obesity, is an additional factor leading to left ventricular hypertrophy [5]. Left ventricular hypertrophy is an independent risk factor for heart failure, coronary heart disease, stroke, arrhythmias, sudden cardiac death, and cardiovascular morbidity and mortality [6].

Population aging may also be characterized by an increasing incidence of frailty [7,8]. Frailty is characterized by increased vulnerability and reduced resilience to different stressors [9] and is another independent risk factor of mortality [9,10]. Several assessment tools are used to define frailty, with the Fried phenotype scale being the first to be developed and widely used in clinical practice and research [9,11,12,13]. The phenotypic frailty concept includes age-associated declines in lean body mass, strength, endurance, balance, and walking performance and low activity [14]. Several papers have documented the relationship between altered cardiac morphology, its function, and frailty [15,16,17]. Nadruz et al. [17] reported that frailty is independently associated with an increased left ventricle mass index. Topriceanu et al. [16] reported that frailty, defined by the accumulation of health deficits (frailty index (FI)), is associated with myocardial hypertrophy and poorer function. The authors of both papers used patients’ weight to index left ventricular mass. Although Nadruz et al. [16] used bioimpedance to assess body composition, they found no association between frailty, obesity, and left ventricular mass. An article by Hamada et al. [18] provides interesting observations regarding frailty, left ventricular systolic function, and heart failure. The authors found that in the elderly Japanese population, the dominant form of heart failure is heart failure with preserved ejection fraction (HFpEF), and the majority of HFpEF patients are frail. The authors suggested that physical frailty is associated with extracardiac factors in HFpEF patients.

Despite growing interest in frailty as a significant factor of worsening prognosis in elderly patients, consistent evidence and proven pathophysiological mechanisms explaining this phenomenon are lacking. Therefore, this study aimed to assess the associations among frailty, lean body mass, central arterial stiffness, and cardiac structure and geometry in older people with a normal ejection fraction.

## 2. Materials and Methods

### 2.1. Study Design and Protocol

This is an ancillary analysis of the FRAPICA study (the ClinicalTrials.org NCT03209414) [19]. This study featured an observational cross-sectional design. Therefore, we followed the Strengthening the Reporting of Observational Studies in Epidemiology (STROBE) checklist to report the methods and findings of this study [20].

### 2.2. Clinical Setting

This study was conducted at the 2nd Department of Cardiology in Zabrze, Medical University of Silesia, Katowice, Poland.

### 2.3. Participants

The study included 205 patients hospitalized between 2017 and 2025 in the 2nd Dept. of Cardiology. The study cohort comprised patients with normal left ventricular function, selected from the entire FRAPICA population of 843 patients enrolled to date. We retrieved medical, laboratory, and echocardiographic data (except the measurements listed below) from patients’ electronic medical records for the index hospitalization period. We did not assess the longitudinal burden of comorbidities, which may constitute residual confounders.

#### 2.3.1. Inclusion Criteria

The study included participants aged > 65 years with informed consent to participate in the project and angiographically confirmed coronary artery disease, in accordance with the FRAPICA trial protocol. Normal left ventricular function, defined as a left ventricular ejection fraction > 50%, was another inclusion criterion for this sub-study.

#### 2.3.2. Exclusion Criterion

The exclusion criterion was a lack of consent to participate in this observational study. Patients with cardiomyopathies, valvular heart disease, previous cardiac surgery, and a left ventricle ejection fraction < 50% were also excluded from the analysis.

### 2.4. Measurements and Variables

We assessed frailty using the Fried frailty phenotype score [9]. We recognized frailty if three or more out of the five following criteria were met:

- Slowness was defined as reduced gait speed over a distance of 5 m. Each patient had to repeat the test three times, and the results were averaged. The results were stratified by gender and height.
- Weakness was assessed with a maximal-handgrip strength test. It was performed with the dominant arm. We used an electronic hand dynamometer, EH101 (VETEK AB, Sweden). The patient was required to perform three repetitions of the procedure, and the maximal value was recorded. The test was positive for frailty if <30 kg for men or <20 kg for women was recorded.
- Low physical activity was assessed by the Minnesota Leisure Time Activity questionnaire. The result was positive when calorie expenditure per week was below 270 kcal/week in women and 383 kcal/week in men. We prepared a Microsoft Excel-based template for rapid questioning and easy calculation of all activities and respective calorie expenditure. We assessed physical activity over the past twelve months.
- Exhaustion was self-reported by each patient. The patient had to answer the following questions from the CESD-R scale: “How often in the past week did you feel like everything you did was an effort? How often in the past week did you feel like you could not get going?” The possible answers were often (3 or more days) or not often, depending on whether the feeling is present for 0–2 days. The positive answer was “often.”
- The last criterion was weight loss exceeding 10 pounds (approximately 4.5 kg) unintentionally in the past year.

Patients meeting 1 or 2 criteria were classified as pre-frail. Patients with a score of 0 were classified as robust.

For the analyses, we pooled the patients classified as robust and pre-frail into a non-frail group.

#### 2.4.1. Assessment of Fat-Free Mass

Patients’ fat-free mass was assessed using Harpenden’s skinfold caliper and Baty’s body assessment software (version 17, Baty International Ltd., Burgess Hill, UK). Lean body mass was derived from a patient’s height and weight using the three-site Jackson/Pollock algorithm [21]. The algorithm uses different measurement sites for men and women (male subjects: chest, abdomen, and thigh; female subjects: triceps, suprailiac, and thigh). The patient’s height and weight were measured using the SECA 284 measuring station (SECA, Hamburg, Germany). Fat-free mass was presented as an absolute value (kg) and as a percentage of total body mass. We internally validated this method against the bioimpedance method with SECA mBCA 528 (SECA, Hamburg, Germany) (see Supplementary Material).

#### 2.4.2. Echocardiographic Assessment

We recorded two-dimensional (2D) images of the left ventricular walls and cavity dimensions at the systole and diastole. Left ventricular mass was measured using 2D M-mode images according to Recommendations for Chamber Quantification of the American Society of Echocardiography and the European Association of Cardiovascular Imaging [22]. Left ventricular hypertrophy was determined if the left ventricular mass index (LVMI) ≥ 115 g/m^2^ in males (reference range: 50–102 g/m^2^) and ≥95 g/m^2^ in females (reference range: 44–88 g/m^2^) [22]. Left ventricular remodeling was determined as normal geometry, concentric remodeling, concentric hypertrophy, and eccentric hypertrophy [22]. The intra-/inter-observer variability was not assessed in this analysis.

#### 2.4.3. Carotid Femoral Pulse Wave Velocity for Assessment of Central Arterial Stiffness

We used carotid–femoral pulse wave velocity (cfPWV) to evaluate central arterial stiffness. For these measurements, we used piezoelectric mechanotransducers at carotid and femoral sites (Complior, Alam Medical, Saint-Quentin-Fallavier, France). This methodology is recommended by the European Society of Hypertension [23]. The right-sided carotid–femoral distance was measured with the Seca mod. 207 height meter (Seca, Hamburg, Germany). Blood pressure was measured in a sitting position, after at least 5 min of rest, using a Microlife BP A1 sphygmomanometer(Microlife AG; Widnau; Switzerland), immediately before PWV assessment, in both arms in triplicate. The measurements were averaged separately for the right and left arms, and the higher value was recorded. Arterial stiffness was expressed in meters per second. The integrated software calculated the central blood pressure from the carotid pulse waveform.

### 2.5. Statistical Analysis

After analyzing the data for normality of distribution and equality of variances using the Shapiro–Wilk test, we applied both parametric and nonparametric statistics to compare non-frail and frail men and women. ANOVA was used for multiple comparisons of normally distributed quantitative data. Any significant differences found in the analysis of variance were further tested with Student’s *t*-test corrected with the Bonferroni method for multiple comparisons. For non-normally distributed data, we used the Kruskal–Wallis ANOVA, with multiple comparisons based on mean ranks. The Chi-square test with Yates’ correction was used to compare frequency data. Data are presented as means and standard deviations or as frequency data. Quantitative data presented are unadjusted, unless indicated otherwise in the manuscript. Spearman’s correlation was calculated to assess the linearity of echocardiographic variables across degrees of frailty. Analysis of covariance (ANCOVA) was used to determine the impact of confounding variables (covariates). The variables that differed significantly in comparative analysis were used as covariates in three ANCOVA models. Model 1 was adjusted for age and gender; model 2 additionally included weight; and model 3 additionally included pulmonary diseases. Statistically significant differences between the analyzed variables were assumed at *p* < 0.05. Statistica v. 13.3, licensed for use by the Medical University of Silesia, was used for all computations.

## 3. Results

### 3.1. Patient Population

Our study population was substantially biased. Frail patients were older in comparison to their non-frail counterparts. Women comprised the majority of the frail group (26 women out of 37 frail individuals, i.e., 70.3%, vs. 63 women out of 168 patients, i.e., 37.5%; *p* < 0.001). The distribution of women’s and men’s morphometric variables (height and weight) was differentiated and affected the mean values of height and weight of non-frail and frail groups (height: 168.6 ± 8.4 vs. 162.4 ± 9.0 cm in non-frail and frail groups, *p* < 0.001; weight: 81.6 ± 14.1 vs. 77.4 ± 14.6 kg in non-frail and frail groups, NS). Frail men and women had comparable weight and height to their non-frail counterparts. The same distribution held for fat-free mass, expressed both as an absolute value and as a percentage of body weight. Body mass index was comparable across all studied groups and subgroups. Likewise, blood pressure at admission and at frailty assessment, central blood pressure, and carotid–femoral pulse wave velocity were comparable between frail and non-frail men and women. Groups were similar in the distribution of comorbidities, except for chronic obstructive pulmonary disease or asthma, which was more common among frail men (Table 1).

### 3.2. Echocardiographic Findings

Left ventricle dimensions differed between men and women. The interventricular septum was insignificantly thicker in men (11.4 ± 1.7 vs. 11.1 ± 1.7 mm, NS) and in frail patients (11.7 ± 2.1 vs. 11.2 ± 1.6 mm, NS). The left ventricle end-diastolic diameter was significantly larger in men (52.5 ± 6.9 vs. 47.3 ± 4.8 mm, *p* < 0.001), and insignificantly smaller in frail patients (48.7 ± 5.8 vs. 50.6 ± 6.7 mm, NS). Posterior wall diameter was comparable in men and women (10.9 ± 1.3 vs. 10.8 ± 1.2 mm, NS). However, it was significantly thicker in frail patients (11.2 ± 1.5 vs. 10.7 ± 1.2 mm, *p* < 0.05). Such trends in left ventricular dimensions resulted in higher, though not statistically significant, left ventricular mass and indexed left ventricular mass in frail patients (left ventricular mass: 217.1 ± 71.7 vs. 214.5 ± 60.0 g in frail and non-frail groups, NS; left ventricular mass index: 116.1 ± 34.2 vs. 110.0 ± 28.6 g in frail and non-frail groups, NS). The relative wall thickness was significantly higher in women than in men (0.46 ± 0.1 vs. 0.42 ± 0.1, *p* < 0.001) and in frail patients (0.47 ± 0.1 vs. 0.43 ± 0.1, *p* < 0.01). Details are presented in Table 2.

**Figure 1:**
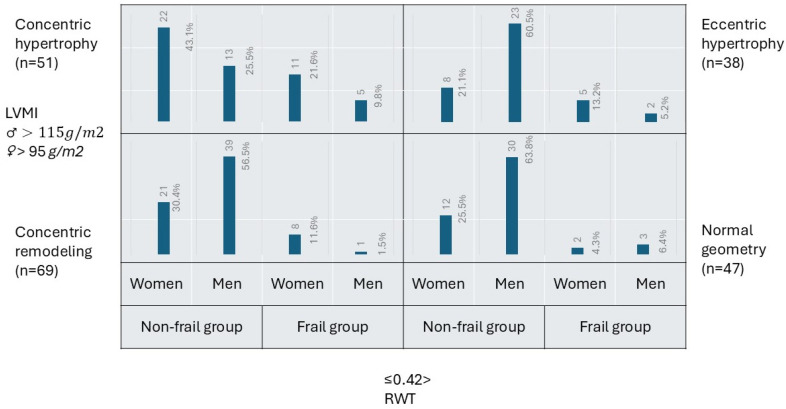
Distribution of left ventricular remodeling based on left ventricular mass index (LVMI) and relative wall thickness (RWT), categorized by gender and frailty status. Numbers represent patients in each category.

Based on the indexed left ventricular mass and relative wall thickness, five frail patients (13.5%) had normal geometry, nine (24.3%) had concentric remodeling, sixteen (43.3%) had concentric hypertrophy, and seven (18.9%) had eccentric hypertrophy. There was a shift in the proportions of men and women among frail patients with different forms of geometry. Women became two to eight times more numerous than men in eccentric, concentric hypertrophy, and concentric remodeling subgroups. Among non-frail patients, most (35.7%) fitted into the concentric remodeling category. The remainder of them were distributed equally among normal geometry, concentric, and eccentric hypertrophy. Women dominated over men in the concentric hypertrophy category. Men comprised the majority of the non-frail population in normal geometry, concentric remodeling, and eccentric hypertrophy categories (Figure 1).

**Figure 2:**
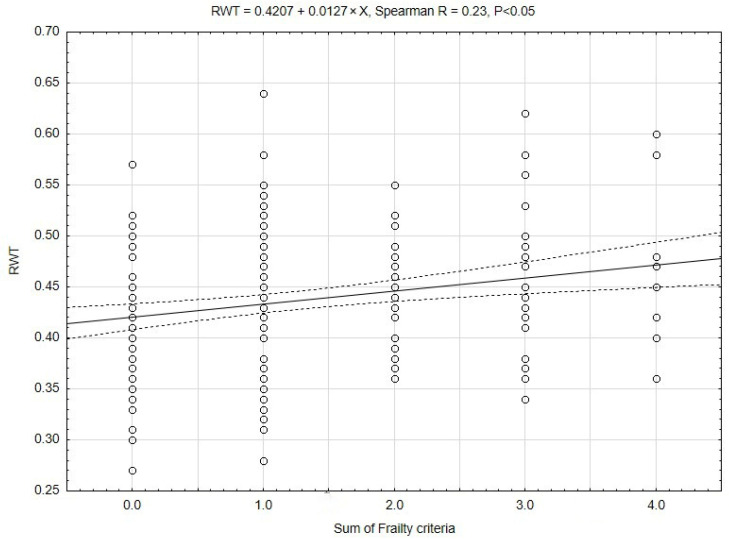
The relationship of the sum of Frailty criteria and relative wall thickness (RWT). The scores 0–2 is for the non-frail group, and the scores 3–4 is for the frail group.

Cardiac remodeling occurs in a linear rather than a binary fashion. To reflect this process, we correlated the left ventricular dimensions, left ventricular mass, and relative wall thickness, as the dependent variables, with the sum of the frailty criteria, as the independent variable (Spearman correlation). The maximal number of frailty criteria observed in our cohort was four. The interventricular septum, posterior wall, left ventricular mass, and indexed left ventricular mass were not correlated. Left ventricle end-diastolic diameter was negatively correlated with the sum of frailty criteria (LVEDD = 51.4 − 0.9 × x; Spearman R = −0.17; *p* < 0.05). A positive correlation was observed with relative wall thickness. This correlation is presented in Figure 2.

### 3.3. Assessment of Covariates

We chose the covariates that differed significantly between the non-frail and frail groups. Adjusting left ventricular dimensions for frailty, gender, age, weight, and pulmonary disease identified patients’ gender and weight as independent covariates. Age was another independent covariate of posterior wall thickness (Table 3). The three above-mentioned variables were independent covariates for left ventricular mass and left ventricular mass index. Frailty and gender were independently related to relative wall thickness.

In a mixed patient population including both men and women, the influence of gender and body weight on specific parameters is evident, especially in a situation when the study cohort is subclassified for frailty. A higher proportion of women is observed in the frail population; therefore, the distribution of body weight shifts downward relative to the non-frail population. Our cohort reflects this observation; moreover, frailty is independently and positively associated with relative wall thickness. To sum up, frailty predisposes women toward concentric remodeling/hypertrophy, with concentric hypertrophy being the dominant type of cardiac remodeling in frail patients.

## 4. Discussion

Frailty, defined by Linda Fried’s phenotype frailty score, in a cohort of hospitalized patients with normal left ventricular function, is independently associated with an adverse cardiac phenotype—one characterized by increased relative wall thickness and concentric remodeling or hypertrophy.

Topriceanu et al. [16] utilized the Frailty Index (FI) as a measure of frailty. The frailty index counts health deficit accumulation as the ratio between the number of deficits present and the total number of deficits appraised [13]. Only one paper compares the Fried criteria and the frailty index [24]. Both methods assess different health and functional domains. Kappa coefficients for the Fried phenotype with FI-28 and FI-40 are 0.357 and 0.408, respectively, indicating fair agreement between the methods. However, the phenotype frailty scale and FI-28 and FI-40 differ in their predictive ability for mortality [24]. Although Topriceanu et al. used a different method to assess frailty in patients, their results are consistent with our findings. They found a positive association between health deficits accumulated over the life course and adverse cardiac remodeling: left ventricular hypertrophy, elevated left ventricular filling pressure, and reduced systolic function. Xi et al. [25] assessed cardiac echo using a cross-sectional approach in a comparable Chinese population (normal left ventricular function; patients’ ages ranged from 70.5 to 76.4 for robust and frail patients, respectively, and the percentage of males decreased across the robust-to-frail groups). The authors used the phenotype frailty scale and found that frailty is independently associated with higher left ventricular mass index, a higher incidence of left ventricular hypertrophy, diastolic dysfunction, and reduced left ventricular longitudinal strain. Ramonfaur et al. [26], based on prospective observations of the ARIC study population, reported that among older adults free of heart failure, progression in frailty and in subclinical left ventricular remodeling and diastolic dysfunction are interrelated and possibly bidirectional. Moreover, they reported that greater left ventricular mass and higher left ventricular filling pressure are associated with greater progression in frailty status, whereas transitioning from robust to prefrail or frail is associated with greater concomitant increases in left ventricular mass and filling pressure.

The frail population is predominantly women, who are lighter and smaller with smaller heart dimensions and lower heart weight. Even after indexing dimensions and the left ventricular mass to body surface area, the normal values for women are lower than those for men. That is why gender should be considered a confounding variable when comparing frail and non-frail populations. We have focused our research on left ventricular mass and geometry, whereas other papers document impaired left ventricular systolic and diastolic function, left atrial enlargement, or right ventricular involvement [16,25,26]. It seems reasonable that frailty affects the entire heart and its function. Still, none of the papers mentioned above document the exact pathophysiologic mechanism underlying the association between frailty and heart involvement. Previous studies on the molecular mechanism of frailty have shown that frailty is accompanied by upregulation of inflammatory cytokines, especially interleukin-6 and C-reactive protein [27,28], a decrease in testosterone level [29], insulin resistance [30], and dysregulation of cytokines involved in bone and muscle metabolism, fibrosis, and vascular wall function [31]. This creates a catabolic milieu in which muscle breakdown exceeds muscle synthesis, leading to a progressive decline in muscle mass and strength (sarcopenia) [32]. Our early working hypothesis was that cardiac muscle is subject to the same processes as skeletal muscle and that frail patients who weigh less have lighter hearts. This was true for younger (18–55 years) patients who were placed on a low-calorie diet for 12 months [31], resulting in weight loss and approximately a 6 g reduction in left ventricular mass as assessed by magnetic resonance imaging. We found a strong positive association between patients’ weight and left ventricular mass, but this appears to be influenced by gender, as the association disappears after indexing for body surface area. The authors of the articles mentioned above [16,25,26] failed either to find an association between body weight and left ventricular mass.

Increased left ventricular afterload, as in hypertension-induced pressure overload, is another trigger for left ventricular hypertrophy. We analyzed blood pressure measured on two occasions (at admission and at frailty assessment), central arterial pressure, and central arterial stiffness, all of which were comparable across all groups. Likewise, the proportion of patients diagnosed with hypertension was similar across the groups. The percentage of hypertension in our cohort ranged from 81 to 92%, which is substantially higher than that reported for the Chinese population (51–53%) by Xi et al. [25]. We did not include blood pressure, central arterial pressure, central arterial stiffness, or hypertension as covariates because their means and distributions were comparable across groups. Nonetheless, Xi et al. [25] reported that frailty and hypertension are independently associated with left ventricular hypertrophy.

While it is true that cardiac muscle does not undergo changes similar to skeletal muscle, we emphasize the importance of concentric remodeling. Our frail patients may be described as those with thicker left ventricular walls and smaller left ventricular cavities than their non-frail counterparts, according to results from linear regression analyses of RWT and LVEDD. An extended analysis of the Multi-Ethnic Study of Atherosclerosis (MESA) population supports our findings [33]. In brief, MESA participants underwent magnetic resonance and computed tomography imaging between 2010 and 2012; subsequently, between 2016 and 2018, they were assessed for frailty and functional capacity. The authors used the modified FRAIL scale, a self-reported questionnaire completed by patients, to stratify them into robust and pooled prefrail/frail subgroups. The authors have found that patients with a reduced end-diastolic volume index (EDVI), an increased mass volume ratio (MVR), and fibrosis (described as extracellular volume) are at increased risk of becoming frail and physically incapacitated with shorter 6 min walking test distance covered [33]. Another ancillary analysis of a MESA population delves deeper into the mechanistic effects of a greater extent of emphysema on CT scanning and pulmonary function on left ventricular end-diastolic volume and reduced left ventricular stroke volume [34]. We report a slightly higher prevalence of pulmonary diseases in our patients than the MESA investigators. Although frail patients were significantly more likely to have pulmonary diseases in our cohort, including this variable in Model 3 ANCOVA did not affect the final results. It seems reasonable to consider this as a hypothesis-generating idea and include the assessment of the extracellular matrix and the mechanistic concept of “shrinking heart” into future research on frailty.

## 5. Limitations of the Study

Our study has several limitations that preclude generalizability of our results to the broader elderly population. We studied a relatively small group of hospitalized patients at a single time point. We did not assess the impact of lifelong exposure to comorbidities, and this should be considered as a residual confounder. We focused on left ventricular mass and geometry, while information on left ventricular diastolic function would serve as a pathophysiological link to heart failure, especially in cases with preserved ejection fraction. Finally, the cross-sectional approach has the least power to infer any causality.

## 6. Conclusions

Frailty is independently associated with left ventricular wall thickening and a reduced left ventricular end-diastolic diameter, resulting in concentric remodeling or hypertrophy. This phenomenon is more pronounced in women. This adverse cardiac remodeling may serve as another phenotype feature of frailty according to the phenotype frailty criteria.

## Figures and Tables

**Table 1:** Demographic data and comorbidities in the study population.

	All Patients (*n* = 205)	Non-Frail Group (*n* = 168)		Frail Group (*n* = 37)		Significance
		Women (*n* = 63) {1}	Men (*n* = 105) {2}	Women (*n* = 26) {3}	Men (*n* = 11) {4}	
Age, years (X ± SD)	72.9 ± 5.1	72.8 ± 4.5	72.2 ± 4.6	75.0 ± 6.1	75.3 ± 7.6	*p* < 0.05; {2} vs. {3} *p* < 0.01
Height, cm (X ± SD)	167.5 ± 8.8	161.0 ± 5.9	173.1 ± 6.1	158.6 ± 6.7	171.3 ± 7.7	*p* < 0.001; {1} vs. {2}, {1} vs. {4} *p* < 0.001
Weight, kg (X ± SD)	80.9 ± 14.3	75.3 ± 14.7	85.4 ± 12.4	76.1 ± 12.8	80.7 ± 18.4	*p* < 0.001; {1} vs. {2} *p* < 0.001; {2} vs. {3} *p* < 0.01
BMI, kg/m^2^ (X ± SD)	28.8 ± 4.2	29.0 ± 4.4	28.5 ± 3.8	30.4 ± 4.9	27.4 ± 4.0	NS
Blood pressure *, mmHg (X ± SD)						
Systolic	142.6 ± 11.1	143.1 ± 12.3	143.2 ± 11.3	141.3 ± 4.9	137.1 ± 12.8	NS
Diastolic	81.0 ± 6.7	82.8 ± 6.8	80.0 ± 6.4	80.4 ± 6.1	81.4 ± 7.6	NS
Blood pressure **, mmHg (X ± SD)						
Systolic	137.6 ± 20.1	140.6 ± 23.6	136.4 ± 17.6	134.7 ± 21.2	137.7 ± 19.9	NS
Diastolic	79.3 ± 10.2	78.9 ± 10.1	79.7 ± 10.2	78.9 ± 10.7	78.4 ± 10.1	NS
Central blood pressure, systolic, mmHg (X ± SD)	130.7 ± 20.9	131.7 ± 26.1	129.6 ± 18.2	131.8 ± 18.2	134.1 ± 20.9	NS
Fat-free mass, kg (X ± SD)	54.6 ± 10.6	47.2 ± 5.8	60.6 ± 8.4	45.3 ± 9.0	60.8 ± 12.0	*p* < 0.001; {1} vs. {2}, {1} vs. {4}, {2} vs.{3}, {3} vs. {4} *p* < 0.001
Fat-free mass, % (X ± SD)	67.8 ± 8.5	63.4 ± 7.1	71.0 ± 7.7	62.7 ± 8.2	75.5 ± 3.9	*p* < 0.001; {1}vs. {2}, {1} vs. {4}, {2} vs.{3}, {3} vs. {4} *p* < 0.001
cfPWV, m/s (X ± SD)	9.9 ± 2.6	9.6 ± 2.7	10.0 ± 2.3	10.0 ± 3.4	9.6 ± 2.2	NS
Comorbidities						
Chronic coronary syndrome, *n* (%)	108 (52.7)	31 (49.2)	58 (55.2)	13 (50.0)	6 (54.5)	NS
Hypertension, *n* (%)	176 (85.9)	53 (84.1)	90 (85.7)	24 (92.3)	9 (81.8)	NS
Diabetes, *n* (%)	73 (35.6)	20 (31.7)	40 (38.1)	8 (30.8)	5 (45.4)	NS
Hyperlipidemia, *n* (%)	162 (79.0)	50 (79.4)	85 (81.0)	19 (73.1)	8 (72.3)	NS
COPD/asthma, *n* (%)	24 (11.7)	6 (9.5)	10 (9.5)	4 (15.4)	4 (36.4)	*p* < 0.05 {2} vs. {4}
Renal failure, *n* (%)	30 (14.6)	5 (7.9)	22 (21.0)	1 (3.8)	2 (18.1)	NS

* blood pressure measured at admission; ** blood pressure measured at frailty assessment; BMI—body mass index; cfPWV—carotid–femoral pulse wave velocity; COPD—chronic obstructive pulmonary disease.

**Table 2:** Echocardiographic assessment of the study population.

	All Patients (*n* = 205)	Non-Frail Group (*n* = 168)		Frail Group (*n* = 37)		Significance
		Women (*n* = 63) {1}	Men (*n* = 105) {2}	Women (*n* = 26) {3}	Men (*n* = 11) {4}	
Left ventricle ejection fraction, % (X ± SD)	62.0 ± 6.4	61.8 ± 6.6	62.2 ± 5.9	61.2 ± 7.2	63.1 ± 7.6	NS
Interventricular septum, mm (X ± SD)	11.3 ± 1.7	11.0 ± 1.8	11.3 ± 1.5	11.2 ± 1.7	12.8 ± 2.6	*p* < 0.05; {1} vs. {4} and {2} vs. {4} and {3} vs. {4} *p* < 0.01
Left ventricle end-diastolic diameter, mm (X ± SD)	50.3 ± 6.6	47.3 ± 4.9	52.6 ± 6.9	47.3 ± 4.6	51.9 ± 7.3	NS
Left ventricle posterior wall, mm (X ± SD)	10.8 ± 1.2	10.7 ± 1.1	11.1 ± 1.3	10.8 ± 1.2	11.5 ± 1.9	NS
Left ventricle mass, g (X ± SD)	215.0 ± 62.1	188.8 ± 45.3	229.9 ± 62.6	196.4 ± 44.1	266.1 ± 99.4	*p* < 0.001; {1} vs. {2} and {1} vs. {4} *p* < 0.001
Left ventricle mass index, g/m^2^ (X ± SD)	111.1 ± 29.7	103.6 ± 24.7	113.8 ± 30.2	107.6 ± 22.6	136.2 ± 48,0	*p* < 0.01; {1} vs. {2} *p* < 0.05, {1} vs. {4} *p* < 0.001, {2} vs. {4} *p* < 0.05, {3} vs. {4} *p* < 0.01
Relative wall thickness, (X ± SD)	0.44 ± 0.1	0.45 ± 0.1	0.42 ± 0.1	0.47 ± 0.1	0.45 ± 0.1	*p* < 0.001; {1} vs. {2} *p* < 0.01; {2} vs. {3} *p* < 0.001

**Table 3:** Analysis of covariance of left ventricular dimensions, mass, mass index, and relative wall thickness with confounding variables as covariates. Model 1 was adjusted for frailty, age, and gender; model 2 was additionally adjusted for weight; and model 3 was additionally adjusted for pulmonary diseases.

Echo Parameter	Covariate	Model 1			Model 2			Model 3		
		β	95% CI	*p*	β	95% CI	*p*	β	95% CI	*p*
IVS	Frailty	−0.13	−0.28–0.01	NS	−0.13	−0.28–0.01	NS	−0.13	−0.27–0.02	NS
	Age	0.08	−0.06–0.22	NS	0.11	−0.03–0.25	NS	0.10	−0.04–0.24	NS
	Gender	−0.15	−0.29–−0.01	*p* < 0.05	−0.10	−0.24–0.05	NS	−0.09	−0.24–0.05	NS
	Weight				0.17	0.02–0.31	*p* < 0.05	0.17	0.02–0.31	*p* < 0.05
	Pulmonary							−0.06	−0.20–0.08	NS
LVEDD	Frailty	0.02	−0.12–0.15	NS	0.02	−0.11–0.15	NS	0.02	−0.11–0.15	NS
	Age	0.03	−0.10–0.16	NS	0.08	−0.05–0.20	NS	0.07	−0.06–0.20	NS
	Gender	−0.39	−0.52–−0.26	*p* < 0.001	−0.30	−0.44–−0.17	*p* < 0.001	−0.30	−0.44–−0.17	*p* < 0.001
	Weight				0.27	0.14–0.41	*p* < 0.001	0.27	0.14–0.41	*p* < 0.001
	Pulmonary							−0.01	−0.14–0.12	NS
PW	Frailty	−0.14	−0.28–0.01	NS	−0.14	−0.28–0.00	NS	−0.13	−0.28–0.01	NS
	Age	0.18	0.04–0.31	*p* < 0.05	0.21	0.07–0.35	*p* < 0.01	0.21	0.07–0.34	*p* < 0.01
	Gender	−0.08	−0.22–0.06	NS	−0.01	−0.15–0.13	NS	−0.01	−0.14–0.13	NS
	Weight				0.21	0.07–0.35	*p* < 0.01	0.21	0.07–0.35	*p* < 0.01
	Pulmonary							−0.01	−0.14–0.13	NS
LVM	Frailty	−0.09	−0.23–0.04	NS	−0.10	−0.22–0.04	NS	−0.10	−0.22–0.04	NS
	Age	0.08	−0.05–0.21	NS	0.13	0.00–0.26	*p* < 0.05	0.13	−0.00–0.26	NS
	Gender	−0.37	−0.50–−0.24	*p* < 0.001	−0.27	−0.41–−0.14	*p* < 0.001	−0.27	−0.40–−0.14	*p* < 0.001
	Weight				0.30	0.18–0.44	*p* < 0.001	0.31	0.17–0.44	*p* < 0.001
	Pulmonary							−0.03	−0.16–0.10	NS
LVMI	Frailty	0.07	−0.24–0.04	NS	−0.10	−0.25–0.04	NS	−0.10	−0.24–0.04	NS
	Age	0.16	0.02–0.29	*p* < 0.05	0.15	0.01–0.29	*p* < 0.05	0.15	0.00–0.29	*p* < 0.05
	Gender	−0.23	−0.37–−0.09	*p* < 0.001	−0.24	−0.39–−0.10	*p* < 0.001	−0.24	−0.38–−0.09	*p* < 0.01
	Weight				−0.04	−0.18–0.10	NS	−0.04	−0.18–0.10	NS
	Pulmonary							−0.03	−0.17–0.11	NS
RWT	Frailty	−0.15	−0.29–−0.01	*p* < 0.05	−0.15	−0.29–−0.01	*p* < 0.05	−0.15	−0.29–−0.01	*p* < 0.05
	Age	0.09	−0.05–0.22	NS	0.07	−0.07–0.20	NS	0.07	−0.07–0.20	NS
	Gender	0.23	0.09–0.36	*p* < 0.01	0.20	0.05–0.34	*p* < 0.01	0.20	0.05–0.34	*p* < 0.01
	Weight				−0.10	−0.24–0.04	NS	−0.10	−0.24–0.04	NS
	Pulmonary							−0.01	−0.14–0.13	NS

IVS—interventricular septum; LVEDD—left ventricular end-diastolic diameter; PW—posterior wall; LVM—left ventricular mass; LVMI—left ventricular mass index; RWT—relative wall thickness; frailty as the reference; gender—male gender as reference; pulmonary—no pulmonary diseases as reference.

## Data Availability

The datasets used in the current study are available from the corresponding author on reasonable request.

## Supplementary Materials

The following supporting information can be downloaded at https://www.mdpi.com/article/10.3390/jcdd13010050/s1. Supplementary Material online—Comparison of the three-point and bioimpedance methods for body composition assessment.
