# Supplementary material for: The Impact of Frailty on Left Ventricle Mass and Geometry in Elderly Patients with Normal Ejection Fraction: A STROBE-Compliant Cross-Sectional Study
Source: J Cardiovasc Dev Dis. 2026 Jan 16;13(1):50. doi: 10.3390/jcdd13010050 (PMC12842445; doi:10.3390/jcdd13010050)

Supplementary materials online

Comparison of the three-point skinfold and bioimpedance analysis of lean body mass.
The bioimpedance equipment was Seca mBCA 528
Five investigators and ten patients (aged 22–92 years) were examined using both methods. The figure below presents the Bland-Altman graph.

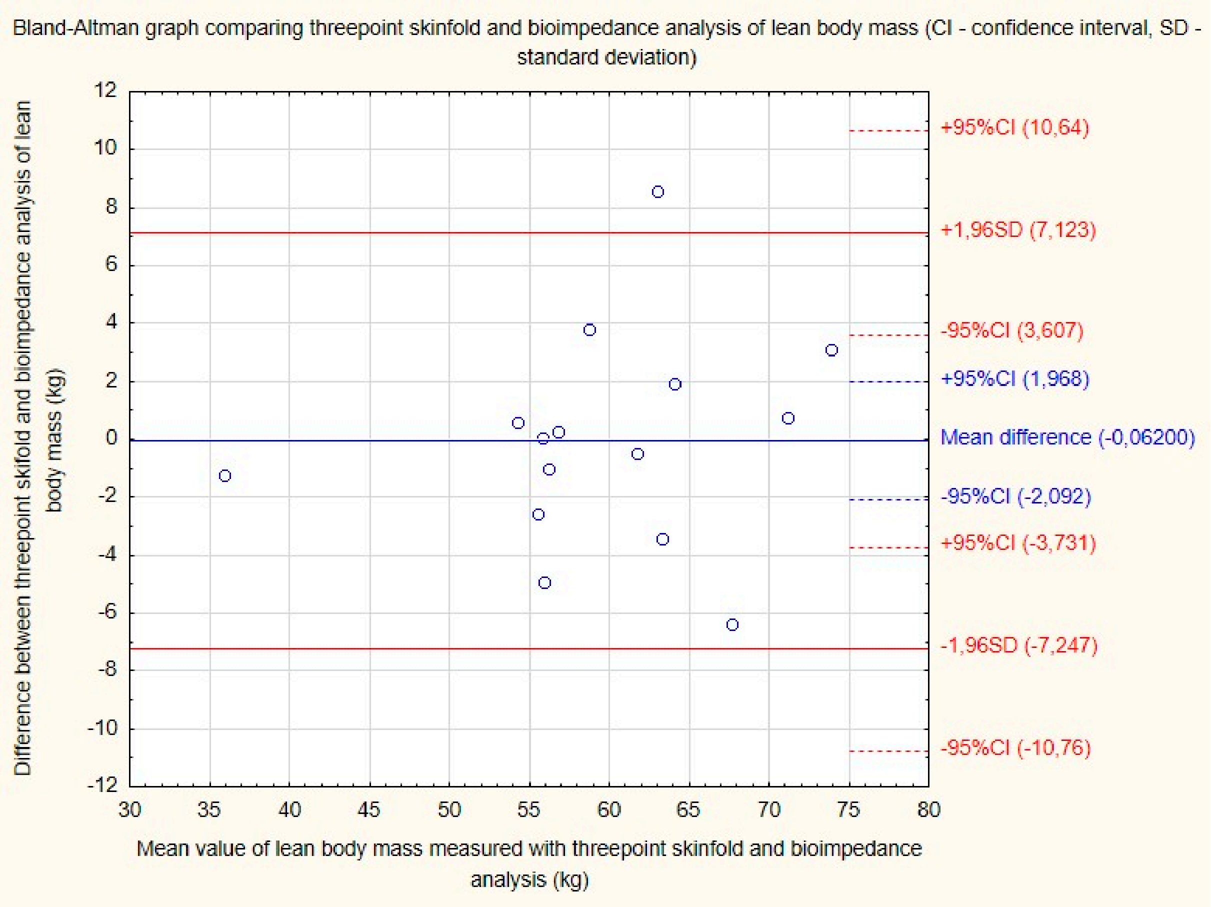

Supplement: Supplementary file 1 [file jcdd-13-00050-s001.zip › jcdd-4084443-supplementary.pdf]
